# Prevalence and Predictors of Adverse Birth Outcomes and Their Implications in Assessing the Safety of New Maternal Vaccines in Kenya

**DOI:** 10.1097/INF.0000000000004660

**Published:** 2025-02-14

**Authors:** Joyce U. Nyiro, Elizabeth Bukusi, Marianne W. Mureithi, David Walumbe, Amek Nyaguara, Collins Kipkoech, Bryan Nyawanda, Godfrey Bigogo, Nancy Otieno, George Aol, Allan Audi, Nickson Murunga, James A. Berkley, D James Nokes, Patrick K. Munywoki

**Affiliations:** * Kenya Medical Research Institute (KEMRI)-Wellcome Trust Research Programme, Centre for Geographic Medicine Research-Coast, Kilifi, Kenya; † Kenya Medical Research Institute (KEMRI), Centre for Microbiology Research, Nairobi, Kenya; ‡ University of Nairobi, Department of Microbiology, Nairobi, Kenya; § Kenya Medical Research Institute (KEMRI), Centre for Global Health Research, Kisumu, Kenya; ¶ Centre for Tropical Medicine & Global Health, University of Oxford, UK; ‖ School of Life Sciences and Zeeman Institute (SBIDER), University of Warwick, Coventry, UK

**Keywords:** Maternal immunization, Adverse birth outcomes, Prevalence, Predictors, Vaccine Safety

## Abstract

**Background:**

Successful introduction, high uptake, and program effectiveness of new maternal vaccines aimed to prevent disease among infants, require prior knowledge of their safety during pregnancy. We aimed to identify background adverse birth outcomes and their predictors in Kenya by which to aid future interpretation of outcomes for new maternal vaccination programs.

**Methods:**

A cross-sectional survey was conducted to assess birth outcomes from women residents within the health and demographic surveillance systems (HDSS) of Kilifi, Siaya and Nairobi, Kenya. All selected women had pregnancies registered in the years 2017 to 2020 through census rounds and had a birth outcome recorded by the time of data collection. These were traced at home for interviews and abstraction of birth outcome records from mother and child health booklets. Multivariable logistic regression was used to identify independent predictors of adverse birth outcomes.

**Results:**

A total of 2,702 women were interviewed. Adverse birth outcomes occurred in 788/2,702 (29.2%) of pregnancies: 433 (16.0%) were preterm (gestational age <37 weeks), 298 (11.0%) low birth weight (<2500g), 99 (3.7%) macrosomic (>4000g) and 41 (1.5%) stillbirths. Predictors of adverse birth outcomes were gestational diabetes (aOR 3.32 (1.53-7.20)), malaria during pregnancy (aOR 1.74 (1.23-2.48)), not attending ANC care (aOR 12.89 (2.17-76.68)) and home delivery (aOR 1.58 (1.18-2.12)).

**Conclusions:**

In three Kenyan settings, almost a third of pregnancies had adverse birth outcomes. Recognizing this baseline prevalence and the factors associated with adverse birth outcomes, will be important in validating safety of new maternal vaccines.

## Background

Recent decades have seen an increase in the development of maternal vaccines that may reduce infant mortality by enhancing immunity in mothers during pregnancy and protecting their babies against infectious diseases in early life[1]. Vaccines against group B Streptococcus (GBS), which is an important cause of stillbirths and preterm births, as well as neonatal invasive bacterial disease, are in final stages of clinical development and have shown promising results[2, 3]. A new maternal respiratory syncytial virus (RSV) vaccine with an efficacy of 82% within the first 90 days of life[4] obtained US Food and Drug Agency (FDA) approval. This vaccine is prioritized for introduction in low-middle income countries (LMICs), where the burden of RSV associated disease is high among infants [1, 5–7]. However, optimal implementation will require a better understanding of the factors influencing these vaccines uptake, including risks, or perception of risks of the vaccine to pregnancy outcomes [8–10].

Assessment of baseline rates of adverse birth outcomes and associated factors ahead of vaccine introduction is likely to benefit interpretation of risks and safety of the new maternal vaccines [10, 11] In clinical trials (NCT04605159;2020-February 2022, NCT04980391;2021-February 2022), to assess an unadjuvanted maternal RSV vaccine in preventing medically attended disease among infants, an increase in number of preterm births was observed in the vaccine arm (238/3496 (6.8%) compared to the placebo arm (86/1739 (4.9%)[14]. Consequently, further trials to this vaccine were suspended[14]. Similarly, the FDA approved maternal vaccine to prevent RSV in infants (Abrysvo), was found protective against severe RSV associated lower-respiratory tract infection (LRTI), but includes a warning to inform of the numerical imbalance in preterm births that occurred in Abrysvo recipients (5.7%) compared to those who received placebo (4.7%) [15]. Since, none of these efficacy trials[4] were conducted in LMICs, safety data of the maternal vaccine among pregnant women and their infants, to guide vaccine rollout and uptake is limited.

Settings with high rates of morbidities such as HIV, malaria and undernutrition are likely to experience the largest burden of adverse birth outcomes, including preterm birth, low birth weight and stillbirth [16, 17], in the absence of an intervention, which may obscure impact of a maternal vaccine program [18]. Many of these birth outcomes are underreported if they do not occur within a health facility [17]. Furthermore, many pregnant women do not complete all recommended antenatal care (ANC) visits[19] where cost-effective interventions to help prevent adverse birth outcomes can be provided.

Using well characterized maternal populations from the HDSS areas of Kilifi, Siaya and Nairobi in Kenya, we quantified the prevalence of adverse birth outcomes, their predictors, and described the implications of these outcomes to aid better understanding of the factors influencing uptake of these vaccines

## Methods

### Study site

This study was conducted within the HDSS areas of Kilifi[20], Siaya[21] and Nairobi [22], in Kenya. The HDSS areas where these data were collected have been described[23]. In brief, the Kilifi HDSS area [20], is situated along the coastal part of Kenya, covering an area of 890km^2^ and a population of ~310,000 residents as of 2022. Kilifi HDSS monitors population through census rounds, three times-a-year, and registers about 8000 pregnancies every year [20]. The KHDSS area had a total of 7,723 and 7,665 births respectively, recorded during enumeration rounds in the years 2017 and 2018. The Kilifi HDSS area is endemic for malaria which has a mortality rate among children aged 6 months to 4 years of 0.57 (95% Confidence Interval [CI] 0.2-1.2) per 1000 person-years [24]. However, in the recent years, malaria incidence has declined, partly due to public health interventions that have reduced transmission [25]. The KHDSS population is served by over 60 health facilities (both private and public) in which pregnant women seek care and about 60% of the deliveries at Kilifi County referral hospital are from this HDSS area [20].

The Siaya and Nairobi sites are managed by Kenya Medical Research Institute (KEMRI)-Centre for Global Health Research (CGHR) with technical and financial support from US Centers for Disease Control and Prevention (CDC)[21]. Both sites are part of a longitudinal Population Based Infectious Disease surveillance (PBIDS) which has been running since 2005. The PBIDS operates in rural western Kenya, Asembo in Siaya County, and in urban informal settlement in Kibera, Nairobi City County. The Asembo PBIDS covers an area of 100 km^2^ with average population of approximately 30,000 (325 people/km^2^) as of December 2018. The area is holoendemic for malaria[26]. In 2018, HIV prevalence in Siaya county was estimated to be 21%, which is among the highest prevalence in Kenya [27]. The Asembo PBIDS area recorded a total of 702 births in 2017, 803 births in 2018 and 818 births in 2019, respectively. The Kibera PBIDS covers two of the 12 villages of the informal settlement, Gatwekera and Soweto West[26]. As of December 2018, approximately 23,000 persons were under follow up in a 0.37 km^2^. [26]. Malaria is not endemic in this area due to the high altitude[28, 29]. Kibera PBIDS area recorded a total of 505 births in 2019, 472 births in 2020 and 504 births in 2021. Since 2015, routine home visits to collect demographic and vital events data such as pregnancy, births, and deaths occur twice a year and the participants receive free medical care for acute infectious illnesses at centrally-located health facility in each site [21].

### Study Population

The study population comprised of women residents registered as pregnant during 2017 through 2020 census rounds in the HDSS areas of Kilifi, Siaya (Asembo) and Nairobi (Kibera). All women had a birth outcome by the time of the interview.

### Study Design

This was a cross-sectional survey to collect data on gestational age at attendance for ANC and birth outcomes among women in Kilifi, Siaya and Nairobi HDSS sites in Kenya.

The target sample size was 1000 women per site[23]. The women were randomly selected from census registers, with an equal number selected from each of the administrative locations [19]. This sample size determination was based on the proportion of women presenting for each ANC visit at a specific gestational age, with a precision of +/-5%, using the median gestational age at first ANC visit of 24 weeks [23], and accounting for availability of ANC booklets for data abstraction [19]. Assuming prevalence of preterm births in the study populations will be like that observed at Kenyatta National Referral Hospital of 18.3% [30], the same sample size of 1000 women, would give a precision of 4.8% in estimating prevalence of preterm births at 95% confidence interval.

To replace women that would be missed during home visits and those with missing booklets as experienced in Kilifi HDSS during data collection, and where only 60% of the selected women were traced[19], the sampling strategy for Siaya and Nairobi sites was modified. An additional random sample of 1000 women, matching the first sample set of 1000 women by geographical location, were selected from each of the census registers of Siaya HDSS and Nairobi PBIDS areas, and the lists uploaded in the study databases and assigned to trained field interviewers for tracing. The replacement list was used if selected women in the first list could not be traced. The interviewers visited homesteads of the selected women, consented them for participation into the study and electronically collected data on ANC attendance, birth outcomes and other obstetric or demographic details using a standardized questionnaire[19].

Data on gestational age at delivery was measured in fundal height and was abstracted from Mother and Child Health (MCH) booklets as recorded by the doctor or midwife. Where records on fundal height were missing, gestational age at birth was computed using the difference between the first date of the last menstrual period (LMP) and the date of delivery, if LMP date was available in the booklet. Exposures assessed were malaria infection during pregnancy, diabetes, pre-eclampsia, urinary tract infections and anemia. These were abstracted from clinicians’ notes as documented in MCH booklets. Socio-demographic characteristics (age, marital status, education level, place of delivery, religion) were abstracted from MCH booklets but if not recorded were obtained through verbal interviews with the participants.

Tracing of participants for interview and data collection for women from Kilifi HDSS began in October 2018 and ended in March 2019. In Asembo PBIDS, Siaya, data collection was conducted from February to May 2021, while in Kibera PBIDS, Nairobi, tracing of participants began in October and ended in December in the year 2021. Data collection in PBIDS sites had been planned for the year 2020 but this was interrupted by the COVID-19 pandemic.

### Ethical considerations

Individual written informed consent was obtained from all the study participants. The study was approved by the KEMRI Scientific and Ethical Review Unit Committee (SERU #3716).

### Statistical Analysis

Data collected from Kilifi, Siaya and Nairobi HDSS sites were merged for analyses of baseline characteristics, birth outcomes and predictors of adverse birth outcomes. These analyses focused on the following poor birth outcomes: stillbirths, preterm births, macrosomia, and low birthweight. A stillbirth was defined as loss of a baby before or during delivery after 20 weeks of pregnancy [31]. Preterm birth (PTB) was defined as baby born alive before 37 weeks of pregnancy were completed [32]. Low birthweight was defined as weight below 2500 grams (g) for a newborn [33]. Macrosomia was defined as infants whose birthweight was over 4,000 grams regardless of gestational age [34].

Proportions, mean (standard deviation: SD) and median (Interquartile range: IQR) were reported in the descriptive analysis. The chi-square test was applied to determine variables associated with choice of a place for delivery and to assess association between adverse birth outcomes and maternal characteristics.

Univariate and multivariable logistic regression models were used to determine predictors for adverse birth outcomes. Maternal age and study site were considered as a priori confounders in the multivariable analysis. Place of delivery, marital status, level of education, occupation, religion, parity, gravida, gestational diabetes, malaria infection, eclampsia, gestational age at delivery, number of ANC visits, timing for ANC initiation and delivery mode were examined in the logistic regression. P-values <0.05 were considered statistically significant. All analysis was conducted in Stata version 15.0 (Stata Corp, College Station, USA).

## Results

### Characteristics of participants

A total of 2,702 women were enrolled: 594 (22.0%) from Kilifi, 1,029 (38.1%) women from Siaya and 1,079 (39.9) from Nairobi. The proportion of births from women that were interviewed, in relation to the total number of births recorded in the study area during the years of sampling were, 3.9% in Kilifi, 43.0% in Siaya and 72.9% in Nairobi. The median age at the time of delivery was 28.9 years (IQR: 24.0-33.4). Of the 2,702 women interviewed, 7 (0.3%) did not attend ANC during pregnancy. Of the 2695 participants who reported to have attended ANC, 2,082 (77.3%) had booklets available to confirm attendance. A total of 1,641 (60.7%) of the women had formal education at primary level, 2,372 (87.8%) were married and 2,585 (95.9%) were Christians (see Table, Supplemental Digital Content 1). Site specific results on characteristics of participants are provided in Table, Supplemental Digital Content 1.

The median birth weight of infants was 3.2 kilograms (kgs) (IQR: 2.9 – 3.5), while estimated median gestational age at delivery was 38.7 weeks (IQR: 36.7 – 40.0), and 265 (9.8%) of births were reported to have occurred at home. The total number of women participants with infants born before 33 weeks of gestation were, 16 (2.7%) Kilifi, 45 (4.4%) Siaya and 10 (0.9%) in Nairobi sites respectively. Vast majority of the women 2,636 (97.2%) interviewed in this study, reported they would accept a new maternal vaccine during ANC visits to prevent pneumonia among their infants (see Table, Supplemental Digital Content 1).

### Prevalence of birth outcomes

Of the 2,702 women interviewed, 788 (29.2%) had adverse birth outcomes; among whom 429 (15.9%) of infants were born preterm <37 weeks’ gestation, 298 (11.0%) were born with low birth weight <2.5 kgs, 99 (3.7%) had macrosomia and 41 (1.5%) were stillbirths (see Table, Supplemental Digital Content 2). Among 77 (2.9%) of the infants born with low birthweight were also preterm.

Proportions with adverse birth outcomes significantly declined from 191/568 (37.8%) in the year 2017 to 323/1074 (30.1%), 177/712 (24.9%) and 85/376 (22.6%) for births occurring in 2018, 2019 and 2020 respectively (p=0.001). Kilifi participants had a significantly higher proportion of adverse birth outcomes compared to Nairobi (38.2% vs 19.9%; p=0.001). The prevalence of adverse birth outcomes was significantly higher for births occurring at home than in hospital (44.9% vs 27.5%; p=0.001) (see Table, Supplemental Digital Content 3).

### Predictors of adverse birth outcomes

Univariate analysis of maternal characteristics and adverse birth outcomes showed significant associations between adverse birth outcomes and lack of formal education (crude Odds Ratio (cOR), 2.08 [95% CI: 1.23-3.52]), home delivery (cOR, 2.15 [95% CI: 1.67-2.79]), not attending ANC during pregnancy (cOR, 8.10 [95 % CI: 1.55-42.10]), gestational diabetes (cOR, 3.51 [95% CI: 1.63-7.55]) and malaria infection during pregnancy (cOR, 1.7 [95% CI: 1.21-2.38]) (see Table, Supplemental Digital Content 3).

In multivariable logistic regression analysis, the independent predictors of adverse birth outcomes were, gestational diabetes (aOR, 3.32 [95% CI: 1.53-7.20]), home delivery (aOR, 1.58 [95% CI: 1.18-2.12]), malaria infection during pregnancy (aOR, 1.74 [95% CI: 1.23-2.48]) and not attending ANC at all during pregnancy (aOR 12.89 [95% CI: 2.17-76.68]) (see Table, Supplemental Digital Content 4). Adjusting for maternal age and HDSS site did not have any effect on the predictors for adverse birth outcomes.

Multivariable logistic regression analysis of specific adverse birth outcomes with maternal characteristics showed predictors of preterm births as, attending one ANC visit or not attending ANC at all (aOR 7.74 [95% CI: 5.38-11.15]), being divorced/widowed or separated (aOR 2.42 [95% CI: 1.23-4.75]), while older maternal age above 30 years was protective of preterm births (aOR 0.54 [95% CI: 0.32-0.90]) (see Table, Supplemental Digital Content 5). The predictors of stillbirths were gestational diabetes (aOR 18.63 [95% CI: 5.21-66.55]) and malaria infection during pregnancy (aOR 5.01 [95% CI: 1.59-15.78]) (see Table, Supplemental Digital Content 6).

## Discussion

In this study we provide a comprehensive description of the prevalence of birth outcomes and the predictors of adverse birth outcomes in three distinct regions in Kenya, ahead of introduction of new maternal vaccines. We found, almost a third (29%) of births in this maternal population had adverse outcomes. The proportion of adverse birth outcomes was significantly higher for births occurring at home than in hospital, with Kilifi (38%) and Siaya (34%) observing higher proportions of adverse birth outcomes compared to Nairobi (20%). Home delivery, gestational diabetes, malaria infection during pregnancy and not attending ANC at all, were found as strong predictors of these adverse birth outcomes. Studies have found individual medical conditions such as gestational diabetes as a danger sign during pregnancy and is likely to increase the risk of premature birth, still births, as well as macrosomia and delivery difficulties in the newborn[35]. Such illness episodes if not well managed may result into poor pregnancy outcomes which might lead to misinterpretation of the outcome of a maternal vaccine.

We found preterm births to be the most prevalent (16%) adverse birth outcomes. Preterm births have been known to have multiple causes [36], and in this study, we found failure to attend ANC and advanced maternal age as strong predictors for preterm births. High rates of preterm births observed during implementation of maternal vaccines programs, in consideration that vaccination in pregnancy with a vaccine such as GBS, might reduce the preterm birth rate, may be considered as a safety signal if background characteristics of that maternal population are unknown. This relates to the observed increased risk of preterm births during phase 3 clinical trials (NCT04605159, NCT04980391, NCT05229068) of a maternal RSV vaccine [13], which resulted into a withdrawal of the vaccine from further clinical trials[37]. In our study, Siaya County is endemic for malaria and is known to have a higher HIV prevalence [27], which may have caused the observed relatively high prevalence of preterm births (4%) occurring within the gestational age period of <33 weeks and other poor pregnancy outcomes in the absence of a new intervention[16]. The prevalence of preterm births was also highest in 2018 and 2021 which we note that, both 2018 and 2021 were preceded by years of restricted access to healthcare services from a long health care workers’ strike in 2017 [38] and restricted movement due to COVID-19 pandemic in 2020, respectively. This may have possibly led to missed ANC visits which is the only platform where pregnant women receive iron and folate supplementation, malaria prophylaxis and are screened for infections such as syphilis and HIV. Mitigating against the observed preterm births in this maternal population, may require at-risk mothers to receive intensified antenatal care during pregnancy.

This study has shown the concern for vaccine hesitancy or refusal of a new maternal vaccine might be minimal in Kenya, as the majority (98%) of women reported they would accept a new maternal vaccine. However, it is worth noting that, perception of risks or beliefs that a new vaccine is likely to cause adverse pregnancy outcomes might result to high rates of vaccine hesitancy or refusals which may affect its uptake[39]. A study conducted in Quebec found the belief that, the A (H1N1) pandemic influenza vaccine had not been adequately tested was associated with lower vaccination rates among the pregnant population[39]. Similarly, following the occurrence of excess preterm births among Abrysvo recipients (5.7%) compared to those who received placebo (4.7%)[15] during phase3 trials, the World Health Organization (WHO) recommended further trials of the maternal RSV vaccine in LMIC’s, to understand its public health value in a population with high rates of comorbidities. These further trials combined with establishment of pregnancy registries for safety surveillance of these new vaccines post-licensure and post-approval, may generate the much-needed knowledge on the safety of the new maternal vaccine in these settings, which will resolve doubts, build confidence, and consequently enhance its uptake among pregnant women when it is rolled out.

The study had some limitations. Sampling of participants from the HDSS register may represent a bias. There were very few participants with data on anemia, pre-eclampsia, hemorrhage, STIs and urinary tract infection recorded at ANC visits resulting to wide confidence intervals. Data on distance from hospital was available for Kilifi participants only. Gestational age at delivery for 77% of women who had MCH booklets in this study, was estimated using fundal height which is not the most accurate measure of gestational age during pregnancy. However, this is what is available and in practice within the public health care system in Kenya. We did not collect data on HIV status among these women. Therefore, the proportions of adverse birth outcomes attributed to HIV infection and how HIV may have altered the observed associations during multivariable analyses could not be ascertained. These data may not be representative of all Kenyan women as it was drawn from a sample of women from three out of the 47 counties in Kenya. However, our sampling covered about 73% of the total births recorded within Kibera PBIDS. The study could also have missed stillbirths that occur in the community since these often go unreported. Nevertheless, the study provides important baseline data on birth outcomes, proportions of adverse birth outcomes and their predictors often missed in studies involving small sample size. The identified factors likely to confound safety outcomes of a new maternal vaccine in this setting, may guide validation of the new maternal interventions during implementation.

## Conclusions

In three Kenyan settings, almost a third of pregnancies had adverse birth outcomes, out of which 16% were preterm births. Births occurring at home were strongly associated with adverse birth outcomes. Vaccine hesitancy may be low in this setting as 98% of women reported willingness to accept uptake of a new maternal vaccine during pregnancy. Recognizing this baseline prevalence will be important in validating safety of a new maternal vaccine.

## Supplementary Material

Supplemental Digital Content (Including Legend)_1

Supplemental Digital Content (Including Legend)_2

Supplemental Digital Content (Including Legend)_3

Supplemental Digital Content (Including Legend)_4

Supplemental Digital Content (Including Legend)_5

Supplemental Digital Content (Including Legend)_6

## Data Availability

The dataset and analysis scripts generated for this manuscript are available in Harvard Dataverse at https://doi.org/10.7910/DVN/9AIEIT. The data is stored under restricted access and available from the authors upon request through submission of a request form http://kemri-wellcome.org/aboutus/#ChildVerticalTab_15 for consideration by our Data Governance Committee (dgc@kemri-wellcome.org).
