# Supplementary material for: Prevalence and Predictors of Adverse Birth Outcomes and Their Implications in Assessing the Safety of New Maternal Vaccines in Kenya
Source: Pediatr Infect Dis J. Author manuscript; Available in PMC 2025 Mar 19. (PMC7617502; doi:10.1097/INF.0000000000004660)
Supplement: Supplemental Digital Content (Including Legend)_1 [file EMS200391-supplement-Supplemental_Digital_Content__Including_Legend__1.docx]

**SUPPLEMENTAL DIGITAL CONTENT 1***.* Characteristics of participating women from Kilifi HDSS, Siaya and Nairobi PBIDS

|  | **Participating Surveillance Sites** | |  |  |  |
| --- | --- | --- | --- | --- | --- |
| **Characteristics** | **Kilifi** | **Siaya (Asembo)** | **Nairobi (Kibera)** | **All participants** |  |
|  |  |  |  |  |  |
|  | **n (%)** | **n (%)** | **n (%)** | **n (%)** |  |
| Total number of births within the study area during years of sampling***** | 15,388 | 2,393 | 1,481 | 19,262 |  |
| Women with births interviewed (N) | 594 (3.9) | 1029 (43.0) | 1079 (72.9) | 2702 (14.0) |  |
| Women attended ANC | 594(100.0) | 1025(99.6) | 1076(99.7) | 2695(99.7) |  |
| Women with ANC booklets | 470 (79.1) | 791 (76.9) | 821 (76.1) | 2082 (77.3) |  |
| Median age (IQR) in years | 28.6(23.4-33.6) | 28.4(24.0-32.5) | 29(25-34) | 28.9(24.0-33.4) |  |
| **Maternal age (years)** |  |  |  |  |  |
| 15-19 | 29 (4.9) | 53 (5.2) | 17 (1.6) | 99 (3.7) |  |
| 20-29 | 275 (46.3) | 509 (49.5) | 548 (50.8) | 1332 (49.3) |  |
| 30-39 | 233 (39.2) | 419 (40.7) | 454 (42.1) | 1106 (40.9) |  |
| 40-49 | 57 (9.6) | 41 (4.0) | 60 (5.6) | 158 (5.9) |  |
| Data not available | 0 (0.0) | 7 (0.7) | 0 (0.0) | 7 (0.3) |  |
| **Education level** |  |  |  |  |  |
| None | 99 (16.7) | 3 (0.3) | 2 (0.2) | 104 (3.9) |  |
| Primary | 423 (71.2) | 674 (65.8) | 544 (50.4) | 1641 (60.7) |  |
| Secondary | 55 (9.3) | 313 (30.5) | 423 (39.2) | 791 (29.3) |  |
| Tertiary-College/University | 17 (2.9) | 35 (3.4) | 110 (10.2) | 162 (6.0) |  |
| Data not available | 0 (0.0) | 4(0.4) | 0 (0.0) | 4 (0.2) |  |
| **Marital status** |  |  |  |  |  |
| Married | 545 (91.8) | 912(89.0) | 915 (85.4) | 2372 (87.8) |  |
| Single | 45 (7.6) | 78(7.6) | 151 (14.0) | 274 (10.1) |  |
| Divorced/Separated/Widowed | 4 (0.7) | 35(3.4) | 10 (0.9) | 49 (1.8) |  |
| Data not available | 0 (0.0) | 4(0.4) | 3 (0.3) | 7 (0.3) |  |
| **Religion** |  |  |  |  |  |
| Christian | 502 (84.5) | 1019(99.4) | 1064 (98.9) | 2585 (95.9) |  |
| Muslim | 59 (9.9) | 6(0.6) | 12 (1.1) | 77 (2.9) |  |
| Other | 33 (5.7) | 0(0.0) | 0(0.0) | 33 (1.2) |  |
| Data not available | 0 (0.00) | 4 (0.39) | 3 (0.28) | 7 (0.26) |  |
| **Median (IQR) Gestational age at First ANC visit in weeks** | 26 (21-28) | 22 (18-26) | 23 (19-26) | 24 (20-28) |  |
| **Median (IQR) Gestational age at delivery in weeks** | 38.0 (36.0-40.0) | 38.4 (36.0-40.0) | 39.1(37.6-40.6) | 38.7(36.7-40.0) |  |
| **Gestational age at delivery** |  |  |  |  |  |
| Early preterm (<33weeks) | 16 (2.7) | 45 (4.4) | 10 (0.9) | 71 (2.6) |  |
| Late preterm (33-36 weeks) | 143 (24.1) | 174 (16.9) | 36 (3.4) | 353 (13.1) |  |
| Term (37-44 weeks) | 430 (72.4) | 609 (59.2) | 673 (62.4) | 1712 (63.4) |  |
| Data not available | 5 (0.8) | 201 (19.5) | 360 (33.4) | 566 (20.9) |  |
| **Parity** |  |  |  |  |  |
| 0 | 98(16.5) | 114 (11.1) | 245 (22.7) | 457 (16.9) |  |
| 1 | 139 (23.4) | 207 (20.1) | 245 (22.7) | 591 (21.9) |  |
| 2-5 | 267(44.95) | 646(62.78) | 529(49.0) | 1442 (53.4) |  |
| >5 | 90 (15.2) | 58 (5.6) | 20 (1.9) | 168 (6.2) |  |
| Data not available | 0 (0.00) | 4 (0.39) | 40 (3.7) | 44 (1.6) |  |
| **Median (IQR) Birth weight (kilograms)** | 3.0 (2.8-3.4) | 3.3 (3.0-3.6) | 3.2 (2.9-3.5) | 3.2 (2.9-3.5) |  |
| **Place of Delivery** |  |  |  |  |  |
| Health facility | 417 (70.2) | 967(94.0) | 1053 (97.6) | 2437 (90.2) |  |
| Home | 177 (29.8) | 62(6.0) | 26 (2.4) | 265 (9.8) |  |
| **Would accept a new maternal vaccine** |  |  |  |  |  |
| Yes | 594 (100.0) | 1015 (98.6) | 1027 (95.5) | 2636 (97.7) |  |
| No | 0 (0.0) | 14 (1.4) | 49 (4.6) | 1. 2.3) |  |

*****Years of sampling for the study in Kilifi HDSS (2017 and 2018), in Asembo PBIDS, Siaya (2017, 2018 and 2019) and in Kibera PBIDS, Nairobi (2019,2020,2021)
