# Supplementary material for: Prevalence and Predictors of Adverse Birth Outcomes and Their Implications in Assessing the Safety of New Maternal Vaccines in Kenya
Source: Pediatr Infect Dis J. Author manuscript; Available in PMC 2025 Mar 19. (PMC7617502; doi:10.1097/INF.0000000000004660)
Supplement: Supplemental Digital Content (Including Legend)_2 [file EMS200391-supplement-Supplemental_Digital_Content__Including_Legend__2.docx]

**SUPPLEMENTAL DIGITAL CONTENT 2.** Prevalence of birth outcomes among women from Kilifi, Siaya and Nairobi in Kenya

|  | **Participating Surveillance Sites** | | |  |  |
| --- | --- | --- | --- | --- | --- |
| **Birth outcomes characteristics** | **Kilifi**  **(N=594)** | **Asembo, Siaya**  **(N=1029)** | **Kibera, Nairobi**  **(N=1079)** | **All participants**  **(N=2702)** |  |
|  |  |  |  |  | **Chi2 P value** |
|  | ***n (%)*** | ***n (%)*** | ***n (%)*** | ***n (%)*** |  |
| **Births** |  |  |  |  |  |
| Normal outcome | 367(61.7) | 683(66.4) | 864 (80.1) | 1914(70.8) | **<0.001** |
| Adverse outcome | 227(38.2) | 346(33.6) | 215 (19.9) | 788 (29.2) |  |
| **Birth weight** |  |  |  |  |  |
| low birthweight <2.5kgs | 92 (15.5) | 88(8.6) | 118 (10.9) | 298 (11.0) | **<0.001** |
| Normal weight >=2.5kgs | 493 (83.0) | 820 (79.7) | 908 (84.2) | 2221 (82.2) |  |
| data not available | 9 (1.5) | 121 (11.8) | 53 (4.9) | 183 (6.8) |  |
| **Preterm births** |  |  |  |  |  |
| Yes (<37 weeks) | 159 (26.8) | 222 (21.6) | 48 (4.5) | 429 (15.9) | **<0.001** |
| No (>=37 weeks) | 432 (7279) | 643 (62.5) | 672 (62.3) | 1747 (64.6) |  |
| Data not available | 3 (0.5) | 164 (15.9) | 359 (33.3) | 526 (19.5) |  |
| **Still births** |  |  |  |  |  |
| Yes | 3 (0.5) | 23 (2.2) | 15 (1.4) | 41 (1.5) | **0.021** |
| No | 591 (99.5) | 1006 (97.8) | 1064 (98.6) | 2661 (98.5) |  |
| **Macrosomia** |  |  |  |  |  |
| Yes | 14 (2.4) | 40 (3.9) | 45 (4.2) | 99 (3.7) |  |
| No | 570 (96.0) | 868 (84.4) | 981 (90.9) | 2419 (89.5) | **<0.001** |
| data not available | 10 (1.7) | 121 (11.8) | 53 (4.9) | 184 (6.8) |  |
