# Supplementary material for: Prevalence and Predictors of Adverse Birth Outcomes and Their Implications in Assessing the Safety of New Maternal Vaccines in Kenya
Source: Pediatr Infect Dis J. Author manuscript; Available in PMC 2025 Mar 19. (PMC7617502; doi:10.1097/INF.0000000000004660)
Supplement: Supplemental Digital Content (Including Legend)_3 [file EMS200391-supplement-Supplemental_Digital_Content__Including_Legend__3.docx]

**SUPPLEMENTAL DIGITAL CONTENT 3.** Factors associated with adverse birth outcomes among women from Kilifi, Siaya and Nairobi in Kenya

| **Adverse birth Outcomes** | | | |  | **Univariate logistic regression** | | |  |
| --- | --- | --- | --- | --- | --- | --- | --- | --- |
|  | **All participants** | **Yes** |  | **Chi2 P value** |  | **Odds Ratio (95%CI)** |  | **P** |
| **Characteristic** | **n** | **n** | **%** |  | **cOR*** |  |  | **value** |
|  | **2702** | **788** | **29.16** |  |  | **LCL** | **UCL** |  |
| **Maternal age** |  |  |  |  |  |  |  |  |
| 15-19 | 99 | 38 | 38.38 |  |  |  |  |  |
| 20-29 | 1332 | 399 | 29.95 | 0.155 |  |  |  |  |
| 30-39 | 1106 | 308 | 27.85 |  |  |  |  |  |
| 40-49 | 158 | 42 | 26.58 |  |  |  |  |  |
| Data not available | 6 | 1 | 14.29 |  |  |  |  |  |
| **Marital status** |  |  |  |  |  |  |  |  |
| Married | 2372 | 676 | 28.50 |  | **Ref** |  |  |  |
| Single | 274 | 88 | 32.12 | **0.019** | 1.19 | 0.91 | 1.56 | 0.189 |
| Divorced/separated/widowed | 49 | 19 | 38.78 |  | 1.59 | 0.89 | 2.84 | 0.119 |
| Data not available | 7 | 5 | 71.43 |  | 6.27 | 1.21 | 32.4 | **0.028** |
| **Education level** |  |  |  |  |  |  |  |  |
| None | 104 | 43 | 41.35 |  | 2.08 | 1.23 | 3.52 | **0.006** |
| Primary | 1641 | 515 | 31.38 | **0.000** | 1.35 | 0.93 | 1.95 | 0.111 |
| Secondary | 791 | 187 | 23.64 |  | 0.91 | 0.62 | 1.34 | 0.650 |
| Tertiary-college/university | 162 | 41 | 25.31 |  | **Ref** |  |  |  |
| Data not available | 4 | 2 | 50.00 |  | 2.95 | 0.4 | 121.63 | 0.287 |
| **Parity** |  |  |  |  |  |  |  |  |
| 0 | 457 | 136 | 29.76 |  |  |  |  |  |
| 1 | 591 | 154 | 26.06 |  |  |  |  |  |
| 2-5 | 1442 | 436 | 30.24 |  |  |  |  |  |
| >5 | 168 | 52 | 30.95 | 0.310 |  |  |  |  |
| Data not available | 44 | 10 | 22.73 |  |  |  |  |  |
| **ANC Initiation** |  |  |  |  |  |  |  |  |
| 0-12 weeks | 512 | 121 | 23.63 |  | **Ref** |  |  |  |
| 13-24 weeks | 769 | 223 | 29.00 |  | 1.32 | 1.02 | 1.71 | **0.034** |
| 25-32 weeks | 510 | 175 | 34.31 | **0.003** | 1.69 | 1.28 | 2.22 | **0.000** |
| 33-42 weeks | 305 | 99 | 32.46 |  | 1.55 | 1.13 | 2.12 | **0.006** |
| Data not available | 606 | 170 | 28.05 |  | 1.26 | 0.96 | 1.65 | 0.094 |
| **Place of delivery** |  |  |  |  |  |  |  |  |
| Hospital | 2437 | 669 | 27.45 |  | **Ref** |  |  |  |
| Home | 265 | 119 | 44.91 | **0.000** | 2.15 | 1.67 | 2.79 | **0.000** |
| **Year of delivery** |  |  |  |  |  |  |  |  |
| 2017 | 508 | 192 | 37.80 |  | **Ref** |  |  |  |
| 2018 | 1081 | 324 | 29.97 | **0.000** | 0.70 | 0.56 | 0.88 | **0.002** |
| 2019 | 716 | 178 | 24.86 |  | 0.54 | 0.43 | 0.7 | **0.000** |
| 2020 | 376 | 86 | 22.75 |  | 0.48 | 0.36 | 0.65 | **0.000** |
| 2021 | 19 | 8 | 42.11 |  | 1.20 | 0.47 | 3.03 | 0.704 |
| **Choice of specific place for delivery** |  |  |  |  |  |  |  |  |
| county referral hospital | 361 | 135 | 37.40 |  | **Ref** |  |  |  |
| sub-county hospital | 268 | 83 | 30.97 | **0.000** | 0.75 | 0.54 | 1.05 | 0.940 |
| health center | 1024 | 242 | 23.63 |  | 0.52 | 0.4 | 0.67 | **0.000** |
| dispensary | 97 | 20 | 20.62 |  | 0.43 | 0.25 | 0.74 | **0.002** |
| private hospital/clinic | 687 | 189 | 27.51 |  | 0.64 | 0.48 | 0.83 | **0.001** |
| Home | 265 | 119 | 44.91 |  | 1.36 | 0.99 | 1.88 | **0.059** |
| **Number of ANC visits** |  |  |  |  |  |  |  |  |
| 0 | 7 | 5 | 71.43 |  | 8.10 | 1.55 | 42.1 | **0.013** |
| 1 | 207 | 79 | 38.16 | **<0.001** | 2.00 | 1.44 | 2.77 | **0.000** |
| 2-4 | 1090 | 343 | 31.47 |  | 1.49 | 1.20 | 1.84 | **0.000** |
| >4 | 746 | 176 | 23.59 |  | **Ref** |  |  |  |
| Data not available | 652 | 185 | 28.37 |  | 1.28 | 1.01 | 1.63 | 0.042 |
| **Gestational diabetes** |  |  |  |  |  |  |  |  |
| Yes | 27 | 15 | 55.56 | **0.000** | 3.51 | 1.63 | 7.55 | **0.001** |
| No | 2081 | 546 | 26.24 |  | **Ref** |  |  |  |
| Data not available | 594 | 227 | 38.22 |  | 1.74 | 1.43 | 2.11 | **0.000** |
| **Malaria** |  |  |  |  |  |  |  |  |
| Yes | 159 | 59 | 37.11 | **0.000** | 1.7 | 1.21 | 2.38 | **0.002** |
| No | 1949 | 502 | 25.76 |  | **Ref** |  |  |  |
| Data not available | 594 | 227 | 38.22 |  | 1.78 | 1.47 | 2.16 | **0.000** |
| **Anemia** |  |  |  |  |  |  |  |  |
| Severe | 13 | 6 | 46.15 |  | 1.93 | 0.65 | 5.78 | 0.239 |
| Moderate | 696 | 211 | 30.32 | **0.001** | 0.97 | 0.98 | 1.15 | 1.150 |
| None | 1506 | 463 | 30.74 |  | **Ref** |  |  |  |
| Data not available | 487 | 108 | 22.18 |  |  |  |  |  |
| *Crude odds ratio | |  |  |  |  |  |  |  |
