# Supplementary material for: Prevalence and Predictors of Adverse Birth Outcomes and Their Implications in Assessing the Safety of New Maternal Vaccines in Kenya
Source: Pediatr Infect Dis J. Author manuscript; Available in PMC 2025 Mar 19. (PMC7617502; doi:10.1097/INF.0000000000004660)
Supplement: Supplemental Digital Content (Including Legend)_4 [file EMS200391-supplement-Supplemental_Digital_Content__Including_Legend__4.docx]

**SUPPLEMENTAL DIGITAL CONTENT 4.** Predictors of Adverse birth outcomes among pregnant women from Kilifi, Siaya and Nairobi in Kenya

|  | |  | |  | |  | |  | |  | |  | |  | |  |
| --- | --- | --- | --- | --- | --- | --- | --- | --- | --- | --- | --- | --- | --- | --- | --- | --- |
| **Adverse birth Outcomes** | | | | | | |  | | **Multivariate logistic regression** | | | | | |  | |
|  | **All Participants** | | **Yes** | |  | | **Chi2 P value** | |  | | **Odds Ratio (95%CI)** | |  | | **P** | |
| **Characteristic** | **n** | | **n** | | **%** | |  |  | **AOR**** | |  |  |  |  | **value** | |
|  | **2702** | | **788** | | **29.16** | |  | |  | | **LCL** | | **UCL** | |  | |
| **Education level** |  | |  | |  | |  | |  | |  | |  | |  | |
| None | 104 | | 43 | | 41.35 | |  | | 1.26 | | 0.71 | | 2.23 | | 0.425 | |
| Primary | 1641 | | 515 | | 31.38 | | **0.000** | | 1.18 | | 0.81 | | 1.73 | | 0.389 | |
| Secondary | 791 | | 187 | | 23.64 | |  | | 0.89 | | 0.6 | | 1.33 | | 0.583 | |
| Tertiary-college/university | 162 | | 41 | | 25.31 | |  | | **Ref** | |  | |  | |  | |
| Data not available | 4 | | 2 | | 50.00 | |  | |  | |  | |  | |  | |
| **Place of delivery** |  | |  | |  | |  | |  | |  | |  | |  | |
| Hospital | 2437 | | 669 | | 27.45 | |  | | **Ref** | |  | |  | |  | |
| Home | 265 | | 119 | | 44.91 | | **<0.001** | | 1.58 | | 1.18 | | 2.12 | | **0.002** | |
| **ANC Initiation** |  | |  | |  | |  | |  | |  | |  | |  | |
| 0-12 weeks | 512 | | 121 | | 23.63 | |  | | **Ref** | |  | |  | |  | |
| 13-24 weeks | 769 | | 223 | | 29.00 | |  | | 1.29 | | 0.97 | | 1.72 | | 0.083 | |
| 25-32 weeks | 510 | | 175 | | 34.31 | | **0.003** | | 1.42 | | 1.03 | | 1.97 | | **0.032** | |
| 33-42 weeks | 305 | | 99 | | 32.46 | |  | | 1.50 | | 1.07 | | 2.09 | | **0.019** | |
| Data not available | 606 | | 170 | | 28.05 | |  | | 0.56 | | 0.31 | | 1 | | 0.051 | |
| **Number of ANC visits** |  | |  | |  | |  | |  | |  | |  | |  | |
| 0 | 7 | | 5 | | 71.43 | |  | | 12.89 | | 2.17 | | 76.68 | | **0.005** | |
| 1 | 207 | | 79 | | 38.16 | | **<0.001** | | 1.70 | | 1.19 | | 2.42 | | **0.003** | |
| 2-4 | 1090 | | 343 | | 31.47 | |  | | 1.24 | | 0.98 | | 1.58 | | 0.077 | |
| >4 | 746 | | 176 | | 23.59 | |  | | **Ref** | |  | |  | |  | |
| Data not available | 652 | | 185 | | 28.37 | |  | | 2.66 | | 1.44 | | 4.89 | | 0.002 | |
| **Gestational diabetes** |  | |  | |  | |  | |  | |  | |  | |  | |
| Yes | 27 | | 15 | | 55.56 | | **<0.001** | | 3.32 | | 1.53 | | 7.2 | | **0.002** | |
| No | 2081 | | 546 | | 26.24 | |  | | **Ref** | |  | |  | |  | |
| Data not available | 594 | | 227 | | 38.22 | |  | | 1.46 | | 1.17 | | 1.82 | | 0.001 | |
| **Malaria** |  | |  | |  | |  | |  | |  | |  | |  | |
| Yes | 159 | | 59 | | 37.11 | | **<0.001** | | 1.74 | | 1.23 | | 2.48 | | **0.002** | |
| No | 1949 | | 502 | | 25.76 | |  | | **Ref** | |  | |  | |  | |
| Data not available | 594 | | 227 | | 38.22 | |  | | omitted | |  | |  | |  | |

**Adjusted odds ratio
