# Supplementary material for: Prevalence and Predictors of Adverse Birth Outcomes and Their Implications in Assessing the Safety of New Maternal Vaccines in Kenya
Source: Pediatr Infect Dis J. Author manuscript; Available in PMC 2025 Mar 19. (PMC7617502; doi:10.1097/INF.0000000000004660)
Supplement: Supplemental Digital Content (Including Legend)_5 [file EMS200391-supplement-Supplemental_Digital_Content__Including_Legend__5.docx]

**SUPPLEMENTAL DIGITAL CONTENT 5.** Predictors of Preterm Births among pregnant women from Kilifi, Siaya and Nairobi in Kenya

| **Adverse birth outcome: Preterm Births** | | | |  | **Multivariate logistic regression** | | |  |
| --- | --- | --- | --- | --- | --- | --- | --- | --- |
|  | **All participants** | **Yes** |  | **Chi2 P value** |  | **Odds Ratio (95%CI)** |  | **P** |
| **Characteristic** | **n** | **n** | **%** |  | **aOR*** |  |  | **value** |
|  | **2702** | **429** | **15.88** |  |  | **LCL**** | **UCL***** |  |
| **Maternal age in years** |  |  |  |  |  |  |  |  |
| 15-19 | 99 | 25 | 25.25 |  | **Ref** |  |  |  |
| 20-29 | 1332 | 226 | 16.97 | **0.005** | 0.67 | 0.41 | 1.11 | **0.121** |
| 30-39 | 1106 | 148 | 13.38 |  | 0.54 | 0.32 | 0.90 | **0.019** |
| 40-49 | 158 | 30 | 18.99 |  | 0.50 | 0.26 | 0.93 | **0.029** |
| Data not available | 7 | 0 | 0.00 |  |  |  |  |  |
| **Education level** |  |  |  |  |  |  |  |  |
| None | 104 | 26 | 25.00 |  | 1.23 | 0.64 | 2.36 | 0.533 |
| Primary | 1641 | 289 | 17.61 | **<0.001** | 1.22 | 0.82 | 1.83 | 0.328 |
| Secondary | 791 | 100 | 12.64 |  | 1.11 | 0.74 | 1.69 | 0.595 |
| Tertiary-college/university | 162 | 11 | 6.79 |  | **Ref** |  |  |  |
| Data not available | 4 | 3 | 75.00 |  |  |  |  |  |
| **Marital status** |  |  |  |  |  |  |  |  |
| Married | 2372 | 372 | 15.68 |  | **Ref** |  |  |  |
| Single | 274 | 44 | 16.06 | **<0.001** | 1.10 | 1.14 | 1.90 | 0.546 |
| Divorced/separated/Widowed | 49 | 10 | 20.41 |  | 2.42 | 1.23 | 4.75 | **0.010** |
| Data not available | 7 | 3 | 42.86 |  |  |  |  |  |
| **Number of ANC visits** |  |  |  |  |  |  |  |  |
| 0-1 | 7 | 3 | 42.86 |  | 7.74 | 5.38 | 11.15 | **<0.001** |
| 2-4 | 1090 | 215 | 19.72 | **<0.001** | 1.66 | 1.27 | 2.17 | **<0.001** |
| >4 | 746 | 96 | 12.87 |  | **Ref** |  |  |  |
| Data not available | 652 | 67 | 1.28 |  | 5.01 | 2.71 | 9.27 | **<0.001** |
| **Place of delivery** |  |  |  |  |  |  |  |  |
| Hospital | 2437 | 350 | 14.36 | **<0.001** | **Ref** |  |  |  |
| Home | 265 | 79 | 29.81 |  | 0.95 | 0.68 | 1.33 | 0.781 |
| **Gestational diabetes** |  |  |  |  |  |  |  |  |
| Yes | 27 | 10 | 37.04 | **<0.001** | **Ref** |  |  |  |
| No | 2081 | 260 | 12.49 |  | 2.29 | 1.02 | 5.12 | 0.044 |
| Data not available | 594 | 159 | 26.77 |  |  |  |  |  |
| **Year of delivery** |  |  |  |  |  |  |  |  |
| 2017 | 508 | 130 | 25.59 |  | 1.13 | 0.82 | 1.56 | 0.467 |
| 2018 | 1081 | 181 | 16.74 | **<0.001** | 1.38 | 1.10 | 1.75 | **0.010** |
| 2019 | 716 | 87 | 12.15 |  | **Ref** |  |  |  |
| 2020 | 378 | 25 | 6.61 |  | 1.01 | 0.73 | 1.4 | 0.949 |
| 2021 | 19 | 6 | 31.58 |  | 5.04 | 1.84 | 13.78 | **0.002** |
| *Adjusted odds ratio **Lower Confidence Limit  ***Upper Confidence Limit |  |  |  |  |  |  |  |  |
