# Supplementary material for: Prevalence and Predictors of Adverse Birth Outcomes and Their Implications in Assessing the Safety of New Maternal Vaccines in Kenya
Source: Pediatr Infect Dis J. Author manuscript; Available in PMC 2025 Mar 19. (PMC7617502; doi:10.1097/INF.0000000000004660)
Supplement: Supplemental Digital Content (Including Legend)_6 [file EMS200391-supplement-Supplemental_Digital_Content__Including_Legend__6.docx]

**SUPPLEMENTAL DIGITAL CONTENT 6.** Predictors of Stillbirths outcomes among pregnant women from Kilifi, Siaya and Nairobi in Kenya

| **Adverse birth outcome: Still Births** | | | |  | **Multivariate logistic regression** | | |  |
| --- | --- | --- | --- | --- | --- | --- | --- | --- |
|  | **All participants** | **Yes** |  | **Chi2 P value** |  | **Odds Ratio (95%CI)** |  | **P** |
| **Characteristic** | **n** | **n** | **%** |  | **aOR**** |  |  | **value** |
|  | **2702** | **41** | **1.52** |  |  | **LCL** | **UCL** |  |
| **Education level** |  |  |  |  |  |  |  |  |
| None | 104 | 2 | 1.92 |  | 6.74 | 0.68 | 67.2 | 0.104 |
| Primary | 1641 | 24 | 1.46 | **0.984** | 1.25 | 0.28 | 5.66 | 0.77 |
| Secondary | 791 | 13 | 1.64 |  | 1.03 | 0.22 | 4.82 | 0.975 |
| Tertiary-college/university | 162 | 2 | 1.23 |  | **Ref** |  |  |  |
| Data not available | 4 | 0 | 0.00 |  |  |  |  |  |
| **Number of ANC visits** |  |  |  |  |  |  |  |  |
| 0-1 | 746 | 3 | 1.40 |  | 3.43 | 0.71 | 16.69 | 0.127 |
| 2-4 | 1090 | 9 | 0.83 | **0.000** | 0.87 | 0.71 | 16.69 | 0.809 |
| >4 | 214 | 7 | 0.94 |  | **Ref** |  |  |  |
| Data not available | 652 | 22 | 3.37 |  | 1.38 | 0.41 | 46.94 | 0.857 |
| **Gestational diabetes** |  |  |  |  |  |  |  |  |
| Yes | 27 | 4 | 14.81 | **0.000** | **Ref** |  |  |  |
| No | 2081 | 34 | 1.63 |  | 18.63 | 5.21 | 66.55 | **0.000** |
| Data not available | 594 | 3 | 0.51 |  | 0.22 | 0.047 | 0.97 | 0.046 |
| **Malaria** |  |  |  |  |  |  |  |  |
| Yes | 159 | 6 | 3.77 | **0.008** | **Ref** |  |  |  |
| No | 1949 | 32 | 1.64 |  | 5.01 | 1.59 | 15.78 | **0.006** |
| Data not available | 594 | 3 | 0.51 |  |  |  |  |  |
| **Delivery Mode** |  |  |  |  |  |  |  |  |
| Spontaneous Vaginal Delivery | 2446 | 33 | 1.35 | **0.002** | **Ref** |  |  |  |
| Cesarean section | 178 | 8 | 4.49 |  | 4.88 | 2.04 | 11.69 | **0.000** |
| Data not available | 78 | 0 | 0.00 |  |  |  |  |  |
| **Adjusted odds ratio |  |  |  |  |  |  |  |  |
